# A Suspected Mosaic 3q29 Deletion in a Patient With Autism Spectrum Disorder and Multiple Micronutrient Deficiencies: A Case Report

**DOI:** 10.1155/crig/9353827

**Published:** 2026-10-22

**Authors:** Shuichi Yatsuga

**Affiliations:** ^1^ Department of Medical Genetics Hakodate Goryoukaku Hospital Hakodate Hokkaido Japan; ^2^ Department of Pediatrics Fukuoka University School of Medicine Fukuoka Japan fukuoka-u.ac.jp

**Keywords:** 3q29 deletion, case report, chromosomal microarray analysis, chromosomal mosaicism, food selectivity, micronutrient deficiency, *NBN*, *RNF168*

## Abstract

**Background:**

3q29 deletion syndrome (MIM #609425) is a rare genomic disorder characterized by variable expressivity and incomplete penetrance. Somatic mosaicism for a 3q29 deletion in an affected proband has rarely been documented. Mosaicism inferred from attenuation of the array signal requires confirmation by an independent method. When a chromosomal deletion encompasses an autosomal recessive (AR) disease gene, evaluation of the second allele may be informative. Extreme food selectivity associated with autism spectrum disorder (ASD) poses substantial but underrecognized nutritional risk.

**Case Presentation:**

An 8‐year‐old Japanese boy with ASD, intellectual disability, absent speech, progressive obesity, hepatosplenomegaly, and congenital toe syndactyly underwent chromosomal microarray analysis (CMA). A 1.27‐Mb interval at 3q29 (arr[GRCh37] chr3:195,804,728–197,074,949) was segmented with a mean log2 ratio of −0.252 across 59 informative probes, against an excellent array quality (derivative log ratio spread 0.179). The signal returned to baseline immediately outside the interval. The magnitude of the attenuation is intermediate between a normal diploid state and that expected for a constitutional heterozygous deletion and is compatible with a suspected mosaic deletion; the laboratory’s automated nomenclature nevertheless assigned a copy number of 2, with the explicit caveat that copy number was not verified, and no independent confirmatory method was applied. The interval is atypical and nested within the recurrent 3q29 region and encompasses *TFRC*, *RNF168*, *PAK2*, and *DLG1*. A targeted 15‐gene DNA repair panel identified no pathogenic or likely pathogenic variant on the second *RNF168* allele. An incidental heterozygous *NBN* variant of uncertain significance (c.265A > G, p.Ile89Val; NM_002485.5) was nondiagnostic and did not alter management. Neither exome nor genome sequencing was available as a reimbursed clinical investigation, and parental samples were not obtained. Microcytosis and hypochromia were present at both time points; serum iron was within the reference interval at baseline and low at follow‐up, ferritin remained within the reference interval throughout, and no threshold‐based criterion for iron deficiency was met. Low 25‐hydroxyvitamin D, low vitamin C, and low retinol‐binding protein were documented at two time points and considered most likely related to the markedly restricted diet. Plasma amino acids; serum carnitine fractions; zinc; selenium; vitamins B1, B2, and B12; immunoglobulins; and alpha‐fetoprotein were unremarkable; serum copper was elevated in the presence of a raised C‐reactive protein, and ceruloplasmin was not measured. The cause of the hepatosplenomegaly remained unresolved.

**Conclusion:**

An attenuated CMA signal at 3q29, compatible with a suspected mosaic deletion but not independently confirmed, was identified in a patient whose phenotype overlaps 3q29 deletion syndrome. Where a deletion encompasses an AR disease gene whose phenotype overlaps the presentation, second‐allele evaluation may add diagnostic resolution. This case highlights the importance of considering nutritional assessment in children with ASD and extreme food selectivity; the biochemical abnormalities persisted at follow‐up, although no vitamin D had been administered before the follow‐up sample and treatment exposure and adherence were not systematically documented.

## 1. Introduction

3q29 deletion syndrome (MIM #609425) is a rare genomic disorder caused by an approximately 1.6 Mb deletion on chromosome 3q29, first characterized by Willatt et al. [1]. Core features include intellectual disability, autism spectrum disorder (ASD), schizophrenia predisposition, and dysmorphic features. Key dosage‐sensitive genes within the region include *PAK2* and *DLG1*, both implicated in neurodevelopmental phenotypes [1, 2]. The syndrome shows marked variable expressivity and incomplete penetrance; although the deletion usually arises de novo, it may be inherited from a parent who is unaffected or only mildly affected, which has direct implications for genetic counseling [2, 3].

Somatic mosaicism for chromosomal deletions arises from postzygotic mitotic errors, resulting in a mixture of deletion‐carrying and euploid cells. On array platforms, mosaic deletions are detected as attenuated log2 ratios. The theoretical log2 ratio for a constitutional heterozygous deletion is −1.0; in practice, signal compression means that observed values on array comparative genomic hybridization platforms typically fall between −0.5 and −0.8. Because the degree of attenuation depends on the platform, the reference sample, and the segmentation algorithm, an attenuated signal alone does not establish a mosaic deletion, and confirmation by fluorescence in situ hybridization, quantitative PCR, multiplex ligation‐dependent probe amplification, or droplet digital PCR is required. Published reports of a proband who is themselves mosaic for a 3q29 deletion are exceedingly rare. Petrin et al. documented somatic mosaicism in a phenotypically unaffected father of a proband with a constitutional 3q29 deletion associated with cleft lip/palate and syndactyly; mosaicism was confirmed by quantitative PCR [3]. No detailed clinical description of a mosaic 3q29 proband has been published.

The 3q29 region also includes *RNF168* (OMIM #612688), encoding an E3 ubiquitin ligase essential for the DNA damage response. Biallelic loss‐of‐function variants in *RNF168* cause RIDDLE syndrome (MIM #611943), an autosomal recessive (AR) disorder reported in only four patients worldwide [4–7]. Core hallmarks include cellular radiosensitivity, combined humoral immunodeficiency (decreased IgG and IgA), elevated alpha‐fetoprotein (AFP), and conjunctival telangiectasia [7]. Where a deletion encompasses an AR disease gene whose phenotype overlaps the patient’s presentation, or where the disorder is clinically actionable, evaluation of the second allele may be considered to assess the possibility of compound heterozygosity.

We report the phenotypic, cytogenomic, molecular genetics, and nutritional findings in an 8‐year‐old Japanese boy in whom an attenuated deletion signal at 3q29 was identified, together with a detailed account of the analytical basis and limitations of that finding.

## 2. Case Report

### 2.1. Ethical Considerations

This case report was approved by the Institutional Review Board of Hakodate Goryoukaku Hospital (Approval No. 2025‐062; date of approval: March 17, 2026). Written informed consent for publication was obtained from the patient’s parents.

### 2.2. Patient History and Presentation

The patient was an 8‐year‐old Japanese male, the fifth child of healthy, nonconsanguineous parents. He was born at 40 weeks and 1 day of gestation via spontaneous vaginal delivery (birthweight 3204 g; length 51 cm; head circumference 33 cm). The neonatal period was unremarkable, with no hypotonia or feeding difficulties. Language acquisition was severely delayed, and no speech developed at any stage. A diagnosis of ASD had been made during the preschool years at another institution; the diagnostic instruments used and the details of that assessment were not available for review. He was unable to participate in standardized cognitive assessment. Intellectual disability was characterized as severe on the basis of parental report and clinical observation; formal psychometric testing was not performed, and no standardized severity classification was available. He was enrolled in a special‐support school and was able to swallow tablets. Congenital syndactyly of the second and third toes of the right foot was surgically corrected at 9 months of age.

Progressive weight gain was noted from age 4 years, with an increase of approximately 10 kg over 12 months. Parents reported no subjective hyperphagia. Dietary assessment revealed extreme food selectivity, with a lifetime repertoire of fewer than 10 foods consisting exclusively of refined carbohydrates and processed items (commercial white bread with margarine or processed ham; sesame‐seasoned rice from school lunches with all side dishes refused; sausages; confectionery). He consistently refused all vegetables, fruits, legumes, fish, eggs, and unprocessed dairy. The absence of snoring and witnessed apneas was noted. He was referred for the evaluation of progressive obesity in the context of ASD and intellectual disability, and the initial assessment was undertaken in August 2025.

### 2.3. Physical Examination

At the initial assessment, in August 2025, height was 124.4 cm (−0.34 SD), weight 38.8 kg (+1.97 SD), BMI 25.1 kg/m^2^ (+2.25 SD), and the Japanese obesity index 61%. At the most recent review, in February 2026 and approximately six months after the initial assessment, height was 127.4 cm (−0.29 SD), weight 43.9 kg (+2.1 SD), and BMI 27.0 kg/m^2^ (+2.3 SD), with an obesity index of 71.2%. There was no thyroid enlargement, striae, acanthosis nigricans, or single palmar crease. Increased lanugo‐like hair density was observed in the mandibular region. Detailed dysmorphological assessment, including documentation of the craniofacial features characteristic of 3q29 deletion syndrome (long narrow face, short philtrum, and prominent ears), was not undertaken, as consent for documentation of facial features was not obtained from the patient’s parents; the presence or absence of these features therefore cannot be commented upon.

Abdominal ultrasonography (performed in November 2025) revealed hepatomegaly (left lobe 153.1 mm, upper limit for age 130.1 mm; right lobe 113.4 mm, upper limit 105.5 mm) with a mild increase in parenchymal echogenicity, a fine parenchymal pattern, a sharp hepatic margin, and a smooth surface. No space‐occupying lesion was seen, and the intrahepatic bile ducts were normal. The spleen was enlarged (spleen index 37.3; upper limit for ages 6–8 years 28.9). The gallbladder, common bile duct, and both kidneys were normal. The pancreas could not be adequately visualized owing to bowel gas and patient movement. No lymphadenopathy, ascites, or pleural effusion was reported. The report noted that respiratory gating was not possible and that hepatic measurements should be regarded as approximate. Urinalysis was normal.

### 2.4. Laboratory Findings

Laboratory findings at the initial assessment in August 2025 and at follow‐up in January 2026, approximately five months later, are presented in Table 1. Microcytosis was present at both time points (MCV 75.2 and 74.7 fL; MCH 24.0 and 22.4 pg) with a normal RDW‐CV. The red‐cell count was 5.21 and 5.53 × 10^6^/μL against a reference interval of 4.0–5.2 × 10^6^/μL: The first value lies at the upper limit, and the second was flagged as raised by the laboratory. Hemoglobin was 12.5 and 12.4 g/dL; these values do not meet criteria for anemia at this age. The iron indices differed between the two time points. At the initial assessment, serum iron was 75 μg/dL and ferritin 17.7 ng/mL, both within the laboratory reference intervals, with a normal CRP (0.06 mg/dL); ferritin lay in the lower part of its range but did not fall below the World Health Organization threshold for depleted iron stores in children aged 5 years and over, which is a serum ferritin below 15 μg/L in the absence of inflammation. At follow‐up, serum iron was low at 30 μg/dL, while ferritin had risen to 46.9 ng/mL against a raised CRP of 1.03 mg/dL; because ferritin is an acute‐phase reactant, that value cannot be taken to reflect iron stores. Transferrin saturation and total iron‐binding capacity were not measured, and hemoglobin electrophoresis was not performed. No threshold‐based criterion for iron deficiency was therefore met at either time point. The Mentzer index (MCV divided by the red‐cell count) was 14.4 at baseline and 13.5 at follow‐up, both above 13, which favors iron deficiency over beta‐thalassemia trait as the explanation for the microcytosis. The red‐cell count was nonetheless at or above the upper reference limit at both time points, a combination more often associated with thalassemia trait than with iron deficiency; since the Mentzer index is a screening heuristic and hemoglobin A2 quantification was not performed, a thalassemia trait cannot be excluded. The findings are accordingly reported descriptively as microcytosis and hypochromia with a low serum iron at follow‐up, a pattern compatible with, but not confirming, iron deficiency.

**TABLE 1:** Laboratory findings at two time points in an 8‐year‐old Japanese boy with a suspected mosaic 3q29 deletion.

Parameter	Initial	Follow‐up	Reference
Hemoglobin (g/dL)	12.5	12.4	> 11.5
Red‐cell count (× 10^6^/μL)	5.21	5.53	4.0–5.2
MCV (fL)	75.2	74.7	80–100
MCH (pg)	24.0	22.4	26–33
RDW‐CV (%)	13.4	13.9	11.5–14.5
Platelets (× 10^4^/μL)	33.5	36.0	15–40
Serum iron (μg/dL)	75	30	60–170
Ferritin (ng/mL)	17.7	46.9	10–60
CRP (mg/dL)	0.06	1.03	< 0.30
Total protein (g/dL)	—	8.5	6.6–8.1
Albumin (g/dL)	4.4	4.5	3.8–5.3
Albumin/globulin ratio	—	1.1	1.2–2.0
AST (U/L)	27	47	10–40
ALT (U/L)	16	35	5–40
ALP, JSCC (U/L)	562	554	age appropriate
γ‐GT (U/L)	12	17	< 30
LDH (U/L)	—	225	age appropriate
Total bilirubin (mg/dL)	0.3	0.3	0.2–1.2
Retinol‐binding protein (mg/dL)	2.0	1.8	2.5–7.0
25‐hydroxyvitamin D (ng/mL)	15.2	9.7	> 20
Vitamin C (μg/mL)	1.9	2.8	4–15
PIVKA‐II (mAU/mL)	—	43	< 40
Vitamin B1 (ng/mL)	—	28	age appropriate
Vitamin B2 (ng/mL)	88.2	96.1	age appropriate
Vitamin B12 (pg/mL)	—	186	age appropriate
Zinc (μg/dL)	—	86	80–130
Copper (μg/dL)	—	174	70–140
Selenium (μg/dL)	13.7	12.1	age appropriate
Magnesium (mg/dL)	—	2.2	1.8–2.6
Total carnitine (μmol/L)	49.7	54.8	45–91
Free carnitine (μmol/L)	44.4	40.8	36–74
Acylcarnitine (μmol/L)	5.3	14.0	6–23
Plasma amino acids, total (nmol/mL)	2916.9	—	2068.3–3510.3
IgG (mg/dL)	—	1643	age appropriate
IgA (mg/dL)	—	154	age appropriate
IgM (mg/dL)	—	132	age appropriate
AFP (ng/mL)	—	1.4	age appropriate
Somatomedin‐C (ng/mL)	128	101	normal for age

*Note*: The initial assessment was performed in August 2025 and follow‐up in January 2026. Reference ranges are age appropriate and are those of the reporting laboratory. An em dash indicates that the analyte was not measured at that time point: PIVKA‐II, lactate dehydrogenase, total protein, the albumin/globulin ratio, vitamins B1 and B12, zinc, copper, magnesium, the immunoglobulins, and alpha‐fetoprotein were measured only at follow‐up, and plasma amino acid analysis was performed only at the initial assessment. AST, aspartate aminotransferase; ALT, alanine aminotransferase; ALP, alkaline phosphatase; γ‐GT, gamma‐glutamyl transferase; LDH, lactate dehydrogenase; PIVKA‐II, protein induced by vitamin K absence or antagonist‐II; AFP, alpha‐fetoprotein; Ig, immunoglobulin; RDW‐CV, red cell distribution width–coefficient of variation.

Abbreviations: CRP, C‐reactive protein; MCH, mean corpuscular hemoglobin; MCV, mean corpuscular volume.

Retinol‐binding protein was low at both time points (2.0 and 1.8 mg/dL; reference 2.5–7.0). Retinol‐binding protein is a surrogate marker influenced by inflammation, hepatic synthetic function, and protein status; serum retinol was not measured, and a diagnosis of vitamin A deficiency cannot be established on this basis. Serum albumin was normal (4.4 and 4.5 g/dL). 25‐hydroxyvitamin D was 15.2 ng/mL at the initial assessment and 9.7 ng/mL at follow‐up, both within the deficient range (the two samples were drawn in late summer and in midwinter, respectively, and a seasonal contribution cannot be excluded), and vitamin C was low at both time points (1.9 and 2.8 μg/mL). PIVKA‐II was 43 mAU/mL against a reference limit of < 40 mAU/mL at follow‐up; it was not measured at the initial assessment; this marginal elevation is suggestive of possible subclinical vitamin K insufficiency and should not be regarded as equivalent to the more pronounced vitamin C and vitamin D abnormalities. Coagulation studies were not performed.

A broader metabolic and nutritional screen was undertaken. Plasma amino acid analysis was essentially normal (total amino acids 2916.9 nmol/mL; reference 2068.3–3510.3), with no pattern suggestive of an inborn error of metabolism. Serum carnitine fractions were within or close to the reference range at both time points (total carnitine 49.7 and 54.8 μmol/L; free carnitine 44.4 and 40.8 μmol/L; acylcarnitine 5.3 and 14.0 μmol/L). Zinc (86 μg/dL), selenium (13.7 and 12.1 μg/dL), magnesium (2.2 mg/dL), and vitamins B1 (28 ng/mL), B2 (88.2 and 96.1 ng/mL), and B12 (186 pg/mL) were all within their reference intervals. Serum copper was elevated at 174 μg/dL (reference 70–140) in the same sample in which CRP was raised at 1.03 mg/dL; ceruloplasmin, serum, and urinary copper studies and slit‐lamp examination for Kayser–Fleischer rings were not performed, and Wilson disease was therefore not formally excluded.

Liver function tests showed AST 27 U/L and ALT 16 U/L at the initial assessment, rising to AST 47 U/L and ALT 35 U/L at follow‐up. Alkaline phosphatase was elevated at 562 and 554 U/L, within the expected range for a child of this age, with a normal *γ*‐glutamyl transferase (12 and 17 U/L) and normal total bilirubin (0.3 mg/dL); lactate dehydrogenase was 225 U/L. Platelet count was normal at both time points (33.5 and 36.0 × 10^4^/μL). Total protein was mildly elevated at 8.5 g/dL with a normal albumin and a low albumin/globulin ratio of 1.1, indicating a relative increase in the globulin fraction. Immunoglobulin levels (IgG 1643 mg/dL, IgA 154 mg/dL, IgM 132 mg/dL) and AFP (1.4 ng/mL) were within normal age‐appropriate ranges. Somatomedin‐C was 128 and 101 ng/mL, normal for age.

### 2.5. Cytogenomic and Molecular Genetic Findings

The etiological evaluation was directed at progressive obesity in a child with ASD, intellectual disability, and absent speech. The differential diagnosis considered at the outset included Prader–Willi syndrome and rapid‐onset obesity with hypothalamic dysfunction, hypoventilation, and autonomic dysregulation (ROHHAD) syndrome, alongside a chromosomal imbalance; chromosomal microarray analysis (CMA) was therefore performed as a first‐tier investigation, together with the metabolic and nutritional screen described above.

CMA was performed by SRL, Inc. (Tokyo, Japan), using the Agilent GenetiSure Dx Postnatal platform with the GenetiSure Dx Postnatal Analysis Method_v1, referenced to GRCh37/hg19 against an Agilent Male Reference. Array quality was excellent, with a derivative log ratio spread (DLRS) of 0.179 (manufacturer’s threshold for “excellent,” < 0.20). Three copy number intervals were reported (Table 2); the karyotype was arr[GRCh37](XY)x1.

**TABLE 2:** Cytogenetic and molecular genetic findings.

Finding	Details	Classification	Interpretation
3q29 interval arr [GRCh37] 3q29 (195804728_197074949)	1270.222 kb; 59 informative probes; mean log2 −0.252; median −0.263; SD 0.209 automated call: x2	Attenuated signal; copy number not verified; not independently confirmed	Compatible with a suspected mosaic deletion; atypical and nested within the recurrent 3q29 region; contains *TFRC*, *RNF168*, *PAK2*, *DLG1*
9p13.1p12 gain arr [GRCh37] 9p13.1p12 (38869901_42416387)x3	3546.487 kb; log2 +0.383	Benign	Pericentromeric euchromatic variant; no OMIM disease genes [8, 9]
11p15.3p15.2 gain arr [GRCh37] 11p15.3p15.2 (12051354_12916367)x3	865.014 kb; log2 +0.567; contains *MICAL2*, *PARVA*, *TEAD1*	VUS, likely benign	No established duplication syndrome; parental testing not obtainable
*RNF168* (second allele)	15‐gene DNA repair panel PID_DDR_v1	No pathogenic/likely pathogenic variant detected	No molecular support for compound heterozygosity or RIDDLE syndrome within the analytical scope of the panel
*NBN*	NM_002485.5:c.511A > G (p.Ile171Val); rs61754966; ToMMo AF 0.00584	VUS (ClinVar: conflicting)	Incidental; nondiagnostic; did not alter management; frequency supports BS1
Exome/genome sequencing	Not performed. Not reimbursed for this indication under the Japanese national health insurance system	—	Not undertaken; pathogenic variants elsewhere in the genome cannot be excluded
Parental CMA	Not performed	—	Parental samples not obtained; de novo versus inherited origin undetermined

*Note*: Chromosomal microarray analysis was performed on the Agilent GenetiSure Dx Postnatal platform (SRL, Inc., Tokyo, Japan); derivative log ratio spread was 0.179. All coordinates are given against GRCh37/hg19.

Abbreviations: AF, allele frequency; CMA, chromosomal microarray analysis; SD, standard deviation; ToMMo, Tohoku Medical Megabank Organization; VUS, variant of uncertain significance.

A 1.27‐Mb interval at 3q29 was segmented (minimum interval chr3:195,804,728–197,074,949, 1270.222 kb; maximum interval chr3:195,753,492–197,101,499, 1348.008 kb) with a reported log2 ratio of −0.256. Probe‐level data were obtained from the laboratory. The interval contains 59 informative CGH probes, with a mean log2 ratio of −0.252 (median −0.263; SD 0.209; range −0.890 to +0.284). Fifty‐four of the 59 probes (91.5%) had negative values. The final probe within the interval (chr3:197,074,890) had a log2 ratio of −0.182, whereas the immediately adjacent probe outside the interval (chr3:197,101,500) had a log2 ratio of +0.007, indicating a sharply demarcated segment boundary rather than a progressive subtelomeric drift. The chromosome and gene views are shown in Figure 1.

**FIGURE 1:**
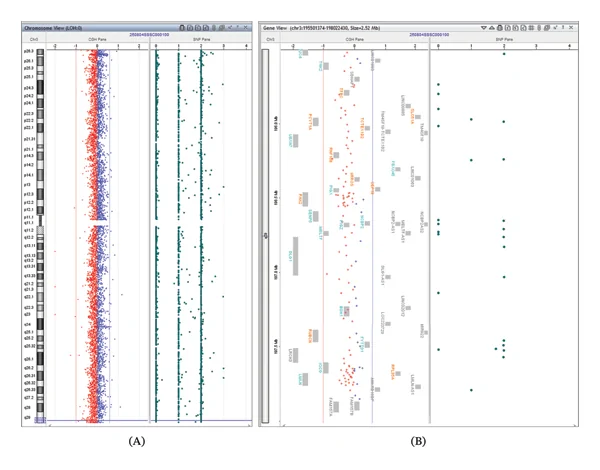
Chromosomal microarray findings at 3q29. (A) Chromosome view of Chromosome 3, showing the CGH pane (left) and SNP pane (right). The segmented interval at 3q29 is indicated at the lower margin. (B) Gene view of the 3q29 region (chr3:195,501,374–198,022,430; GRCh37, 2.52 Mb), showing individual probe log2 ratios and the genes encompassed by the interval, including *TFRC*, *RNF168*, *PAK2*, and *DLG1*. Analysis was performed on the Agilent GenetiSure Dx Postnatal platform; derivative log ratio spread was 0.179. The attenuated signal shown here was not confirmed by an independent method. Data provided by SRL, Inc., Tokyo, Japan.

These probe‐level values are presented descriptively. Adjacent array probes within a segmented interval are not independent biological observations, and inferential statistical testing of the segment mean was therefore not undertaken; such testing would not, in any event, substitute for confirmation by an independent laboratory method.

Assuming an abnormal cell fraction *f*, the expected log2 ratio is log2 ((2 − *f*)/2). Applying this relationship to the observed mean of −0.252 yields *f* ≈ 32%. If allowance is made for the signal compression typical of array CGH, whereby a constitutional heterozygous deletion yields approximately −0.5 to −0.7 rather than the theoretical −1.0, the corresponding estimates are 36%–50%. These figures therefore span approximately 32%–50%. They are platform‐ and algorithm‐dependent inferences derived from signal intensity and do not constitute a direct measurement of the proportion of abnormal cells; the mosaic fraction was not independently determined. The laboratory’s automated ISCN nomenclature assigned a copy number of 2 to this interval (arr[GRCh37] 3q29(195753492x2,195804728_197074949x2,197101499x2)), with the explicit caveat that copy number was not verified; on enquiry, the laboratory confirmed that this assignment was generated automatically by the analysis software and that the underlying threshold criteria were not available to them.

The interval is smaller than the canonical recurrent 3q29 deletion and is nested within it, with the proximal breakpoint lying inside the recurrent region; it is therefore an atypical, nested interval. The genes annotated within the interval are *TFRC*, *LINC00885*, *ZDHHC19*, *SLC51A*, *PCYT1A*, *TCTEX1D2*, *TM4SF19*‐*TCTEX1D2*, *TM4SF19*, *TM4SF19*‐*AS1*, *UBXN7*, *UBXN7*‐*AS1*, *RNF168*, *SMCO1*, *WDR53*, *FBXO45*, *LINC01063*, *NRROS*, *CEP19*, *PIGX*, *PAK2*, *SENP5*, *NCBP2*, *NCBP2*‐*AS1*, *NCBP2AS2*, *PIGZ*, *MELTF*, *MELTF*‐*AS1*, *DLG1*, *MIR4797*, and *DLG1*‐*AS1* (Figure 1B).

Two copy number gains were also identified. A 3.55‐Mb gain at 9p13.1p12 (arr[GRCh37] 9p13.1p12(38869901_42416387)x3; log2 +0.383) lies within the pericentromeric euchromatic region and contains no OMIM disease genes; gains of this region are recurrently reported in phenotypically normal individuals and were classified as benign [8, 9]. A 0.87‐Mb gain at 11p15.3p15.2 (arr[GRCh37] 11p15.3p15.2(12051354_12916367)x3; log2 +0.567) contains *LINC02547*, *MICAL2*, *MIR6124*, *MICALCL*, *PARVA*, and *TEAD1*. No established duplication syndrome involves this interval, and it was classified as a variant of uncertain significance, likely benign. Parental testing was not obtainable. No regions of homozygosity were detected.

Because the interval encompassed *RNF168*, a targeted 15‐gene DNA repair panel (PID_DDR_v1; Kazusa DNA Research Institute, Kisarazu, Japan; genes: *ATM*, *MRE11*, *NBN*, *RAD50*, *LIG4*, *NHEJ1*, *DCLRE1C*, *PRKDC*, *DNMT3B*, *ZBTB24*, *CDCA7*, *HELLS*, *RNF168*, *MCM4*, *BLM*) was performed to assess whether a pathogenic variant on the second *RNF168* allele could create compound heterozygosity. This targeted approach was prioritized because RIDDLE syndrome is clinically actionable—cellular radiosensitivity has direct implications for the use of ionizing radiation—and because its cardinal laboratory features, humoral immunodeficiency and an elevated AFP, could be tested directly and concurrently. The panel covers coding exons and flanking intronic sequence; it does not reliably detect deep intronic variants, variants in regulatory regions, or intragenic copy number changes. No pathogenic or likely pathogenic *RNF168* variant was detected within the analytical scope of the panel, providing no molecular support for compound heterozygosity or RIDDLE syndrome. Normal immunoglobulin and AFP levels were consistent with this.

An incidental heterozygous variant was identified in *NBN*: c.265A > G (p.Ile89Val; NM_002485.5; rs61754966), classified as having conflicting interpretations of pathogenicity in ClinVar. The allele frequency in the Japanese ToMMo database is 0.00584, substantially higher than would be expected for a pathogenic allele causing a disorder of the rarity of Nijmegen breakage syndrome, supporting ACMG criterion BS1. This variant was nondiagnostic and did not alter clinical management; any decision regarding surveillance would rest on the patient’s personal and family history rather than on this finding.

Clinical exome or genome sequencing was not undertaken. At the time of evaluation, the attenuated 3q29 signal, in conjunction with the phenotype, was considered to provide a candidate explanation, and the immediately actionable question—whether a second *RNF168* allele created compound heterozygosity for a clinically actionable AR disorder—was better addressed by the targeted panel. The scope of the genetic evaluation was also constrained by reimbursement. Under the Japanese national health insurance system, neither clinical exome nor genome sequencing is reimbursed for this indication, whereas the targeted DNA repair panel, which includes *RNF168*, was available as a covered investigation. Broader sequencing was therefore not offered to the family; it would be available only on a self‐funded or research basis, it remains an option should further evaluation be sought, and its absence is acknowledged as a limitation. The possibility of an asymptomatic or only mildly affected parent was explicitly considered and family testing was raised for discussion, in view of the incomplete penetrance and variable expressivity of 3q29 deletion syndrome [2, 3]; however, parental samples were not obtained and parental CMA could not be performed. The reason the samples were not obtained is not recorded in the clinical notes: Family testing was raised for discussion, but no decision and no declinature were documented. The de novo versus inherited origin of the interval therefore remains undetermined, and recurrence‐risk counseling remains incomplete.

### 2.6. Management and Outcomes

Vitamin A supplementation was initiated at 10,000 IU/day orally, accompanied by iron supplementation and dietary counseling to enhance vitamin C intake through commercially available vitamin C–fortified confectionery, this being the only vehicle the patient would reliably accept. A plan for vitamin D supplementation was established. Referrals were made for ophthalmological, dental, and cardiac evaluation. The patient was referred to an obesity outpatient clinic and to a registered dietitian with expertise in ASD‐associated feeding difficulties. The family independently commenced a commercial sulforaphane supplement.

Repeat laboratory testing in January 2026, approximately five months after the initial assessment, showed persistent biochemical abnormalities. Retinol‐binding protein was essentially unchanged (2.0–1.8 mg/dL) during vitamin A supplementation; 25‐hydroxyvitamin D fell from 15.2 to 9.7 ng/mL, although no vitamin D had been administered before the follow‐up sample was drawn—only a supplementation plan had been established—and that sample was taken in midwinter; vitamin C rose from 1.9 to 2.8 μg/mL but remained below the reference range; and serum iron fell from 75 to 30 μg/dL, with a rise in ferritin that was confounded by a concurrent rise in CRP. The duration of exposure to vitamin A and iron supplementation, and adherence to them, were not systematically documented; these observations therefore indicate that the abnormalities persisted and do not establish that supplementation failed. Anthropometry at the most recent review, in February 2026 and approximately six months after the initial assessment, showed continued weight gain, with the obesity index increasing from 61% to 71.2% and BMI from 25.1 to 27.0 kg/m^2^. The patient’s parents reported some broadening of accepted foods following the dietitian referral. However, the number and types of newly accepted foods, the duration of follow‐up, and any structured measure of dietary intake were not systematically documented; this parental report is not corroborated by the biochemical or anthropometric data and should be regarded as anecdotal. Parental CMA was not obtainable.

## 3. Discussion

This report describes an attenuated CMA signal at 3q29, compatible with but not establishing a mosaic deletion, in a patient whose phenotype overlaps 3q29 deletion syndrome, together with a systematic evaluation of the second allele of an AR disease gene contained within the interval and a detailed nutritional assessment.

The mean log2 ratio of −0.252 across 59 informative probes is intermediate between a normal diploid state and the value expected for a constitutional heterozygous deletion. Three descriptive observations bear on the interpretation. First, the array quality was excellent (DLRS 0.179), so the attenuation is unlikely to reflect poor hybridization performance. Second, the attenuation was consistent across the interval, with 91.5% of probes negative. Third, the signal returned to baseline immediately outside the segment boundary, whereas a systematic subtelomeric artifact would be expected to produce a progressive drift toward the chromosome end. These observations are, however, all derived from the same CMA experiment and cannot independently establish a deletion. We therefore regard a mosaic deletion as a plausible explanation for the attenuated signal, while recognizing that the finding remains unconfirmed and is presented throughout as suspected rather than established.

The only previously documented instance of somatic mosaicism in the context of 3q29 deletion involved a phenotypically unaffected father whose mosaic state was identified only because of his proband child with a constitutional deletion [3]; in that report, mosaicism was confirmed by quantitative PCR, an independent method that was not available in the present case. That individual was clinically asymptomatic, whereas the present patient exhibits a pronounced neurodevelopmental phenotype. Given the well‐recognized variable expressivity and incomplete penetrance of 3q29 deletion syndrome [2, 3], a mosaic state should not be presumed to confer a uniformly mild phenotype.

Several features of the present patient are consistent with published descriptions of 3q29 deletion syndrome, including developmental delay and intellectual disability, ASD, and feeding difficulties [1, 2]. Other features diverge. Obesity contrasts with the low weight and failure to thrive more commonly reported in this condition, and in the present patient, this is more plausibly attributable to a calorie‐dense, nutritionally narrow diet than to the 3q29 finding. Hepatosplenomegaly is not a recognized feature and is not attributed to it. Toe syndactyly has been reported only occasionally. The craniofacial phenotype could not be assessed for the reasons given above, and psychiatric outcomes including the reported schizophrenia predisposition cannot be evaluated at this age. The interval identified here is atypical and nested within the recurrent region, which may itself bear on the phenotype. Given the uncertainty regarding the presence of a deletion, the copy number state, and the mosaic fraction, these comparisons are presented descriptively and no genotype–phenotype conclusions are drawn.

The 3q29 interval encompassing *RNF168* prompted evaluation of the second allele. RIDDLE syndrome, caused by biallelic *RNF168* loss of function, affects only four patients worldwide [4–7] and is characterized by immunoglobulin deficiency, elevated AFP, and cellular radiosensitivity. Immunoglobulins and AFP were normal in this patient. Cellular radiosensitivity was not assessed—no functional or cytogenetic radiosensitivity testing was undertaken—and is therefore reported as unassessed rather than absent. The panel identified no pathogenic or likely pathogenic variant on the second allele within its analytical scope. We suggest that second‐allele evaluation may be considered where a deletion encompasses an AR disease gene whose phenotype overlaps the patient’s presentation, or where the disorder is clinically actionable, rather than as a routine step in all such cases. We emphasize that a targeted panel addresses only the gene or genes selected; where the phenotype is not fully accounted for, exome or genome sequencing remains the more comprehensive investigation, and it was not performed here.

Hepatomegaly with increased parenchymal echogenicity and disproportionate splenomegaly were notable findings. Hepatosplenomegaly is not a recognized feature of 3q29 deletion syndrome [1, 2], and we do not attribute it to the 3q29 finding. A normal platelet count at both time points, together with normal albumin and bilirubin, argues against portal hypertension. Subsequent investigation was also unrevealing: Plasma amino acids and serum carnitine fractions were normal, arguing against an amino acid disorder or a systemic carnitine deficiency; immunoglobulins and AFP were normal, arguing against a lymphoproliferative process and against RIDDLE syndrome; and a normal *γ*‐glutamyl transferase and bilirubin argue against a cholestatic process. A mildly raised total protein with a low albumin/globulin ratio indicates a relative increase in the globulin fraction, which is nonspecific. Serum copper was elevated in a sample with a concurrently raised CRP; because ceruloplasmin is an acute‐phase reactant, this pattern is most readily explained by inflammation, but ceruloplasmin, urinary copper, and slit‐lamp examination were not performed and Wilson disease has therefore not been formally excluded. Steatotic liver disease secondary to the patient’s dietary pattern is one possibility, and the rise in transaminases between the two time points is consistent with this; however, an occult hematolymphoid process and a subclinical lysosomal storage disorder cannot be excluded. Coagulation studies, portal Doppler assessment, and lysosomal enzyme assays were not performed. The cause of the hepatosplenomegaly therefore remains unresolved, and ceruloplasmin with 24‐h urinary copper, portal Doppler ultrasonography, and lysosomal enzyme assays are recommended.

The patient showed concurrent abnormalities across several micronutrient pathways: microcytosis and hypochromia with a low serum iron at follow‐up, marked 25‐hydroxyvitamin D deficiency, low vitamin C, low retinol‐binding protein, and a marginal elevation of PIVKA‐II. These were considered most likely related to the markedly restricted diet; a broader screen of amino acids, carnitine, zinc, selenium, magnesium, and B‐group vitamins was unremarkable, indicating that the deficiencies were selective rather than global. Food selectivity is a well‐documented comorbidity in ASD, with prevalence rates of 46%–89% [10]; the degree of restriction in this patient, with a lifetime repertoire of fewer than 10 foods, was exceptional. Vitamin D deficiency is of particular concern given its reported associations with adverse neurodevelopmental outcomes in ASD [11]. We note that *TFRC*, which encodes transferrin receptor 1, lies at the proximal end of the interval; this is presented as an observation only, and no causal relationship with the iron indices is claimed. At follow‐up, these abnormalities persisted. Because treatment exposure and adherence were not systematically documented, and because no vitamin D had been given before the second sample was drawn, this cannot be read as a failure of supplementation. It does suggest that such abnormalities are unlikely to resolve while the underlying dietary restriction is unchanged, and it supports early and structured involvement of a dietitian experienced in ASD‐associated feeding difficulties alongside supplementation. The principal message of this case is that concurrent abnormalities across multiple micronutrient pathways may accumulate silently in children with extreme food selectivity, and that it highlights the importance of considering nutritional assessment—iron studies with ferritin and CRP, fat‐soluble vitamins, and vitamin C—in this population. A single case cannot establish the value of systematic screening, and no such claim is made.

Several limitations warrant acknowledgment. First, and most importantly, the 3q29 finding rests entirely on a single CMA experiment. The array plots, the probe‐level values, and the segment‐boundary observation are all derived from that experiment and do not independently confirm a deletion. Orthogonal confirmation by fluorescence in situ hybridization, quantitative PCR, multiplex ligation‐dependent probe amplification, or droplet digital PCR was not performed and was no longer feasible in the present clinical setting. The laboratory’s automated nomenclature assigned a copy number of 2, with the explicit caveat that copy number was not verified. The copy number state therefore remains unconfirmed. Second, the estimated mosaic fraction is a platform‐ and algorithm‐dependent inference from signal intensity rather than a direct measurement of abnormal cells and was not independently determined. Third, parental samples were not obtained and parental CMA was not obtainable, and the de novo versus inherited origin of the interval is undetermined. Fourth, exome or genome sequencing was not performed, neither being reimbursed for this indication under the Japanese national health insurance system, so pathogenic variants elsewhere in the genome, including those that might account for the hepatosplenomegaly, cannot be excluded. Fifth, the estimate applies to peripheral blood leukocytes and may not reflect other tissues, particularly the central nervous system. Sixth, detailed dysmorphological assessment was not undertaken because consent for documentation of facial features was not obtained. Seventh, the ASD diagnosis was established elsewhere and the diagnostic instruments used were unavailable for review; intellectual disability was characterized as severe on the basis of parental report and clinical observation rather than formal psychometric testing. Eighth, follow‐up comprised two time points over approximately 5 months; the reported broadening of the dietary repertoire rests on parental report alone and was not corroborated by any structured dietary measure; the duration of, and adherence to, supplementation was not documented, and no vitamin D was administered before the follow‐up sample, so the persistence of the biochemical abnormalities cannot be attributed to failure of treatment. Ninth, transferrin saturation, total iron‐binding capacity, serum retinol, and coagulation studies were not measured; hemoglobin electrophoresis and hemoglobin A2 quantification were not performed; PIVKA‐II was measured only at follow‐up; cellular radiosensitivity was not assessed; and ceruloplasmin, urinary copper, portal Doppler assessment, and lysosomal enzyme assays were not undertaken.

In conclusion, an attenuated CMA signal at 3q29 was identified in an 8‐year‐old boy with ASD and intellectual disability. The magnitude of the attenuation is compatible with a suspected mosaic deletion, but the finding was not confirmed by an independent method and the copy number state remains unverified; it should not be regarded as establishing a mosaic deletion. Where a deletion encompasses an AR disease gene whose phenotype overlaps the presentation, evaluation of the second allele may add diagnostic resolution; where the phenotype remains incompletely explained, broader sequencing should be considered. Extreme ASD‐associated food selectivity was accompanied by concurrent abnormalities across several micronutrient pathways, which highlights the importance of considering nutritional assessment in children with ASD and extreme food selectivity. The hepatosplenomegaly remained unexplained after the investigations reported here and is not attributed to the 3q29 finding.

## Author Contributions

Shuichi Yatsuga: conceptualization, clinical care, data curation, formal analysis, writing–original draft, and writing–review and editing.

## Funding

The author has nothing to report.

## Disclosure

The author has read and agreed to the published version of the manuscript.

## Conflicts of Interest

The author declares no conflicts of interest.

## Supporting Information

Additional supporting information can be found online in the Supporting Information section.

## Supporting information


**Supporting Information** CARE_Checklist_9353827_R5.

## Data Availability

The data supporting the findings of this study can be obtained from the corresponding author upon reasonable request in accordance with institutional privacy and consent restrictions.
