# Supplementary material for: A Suspected Mosaic 3q29 Deletion in a Patient With Autism Spectrum Disorder and Multiple Micronutrient Deficiencies: A Case Report
Source: Case Rep Genet. 2026 Oct 22;2026:9353827. doi: 10.1155/crig/9353827 (PMC13688419; doi:10.1155/crig/9353827)
Supplement: Supplementary file 1 [file CRIG-2026-9353827-s001.docx]

**CARE CHECKLIST**

2013 CARE Guidelines for Case Reports

**Manuscript 9353827 — Case Reports in Genetics**

**A Suspected Mosaic 3q29 Deletion in a Patient with Autism Spectrum Disorder and Multiple Micronutrient Deficiencies: A Case Report**

Corresponding author: Shuichi Yatsuga, MD, PhD | ORCID: 0000-0002-4064-3102

Department of Medical Genetics, Hakodate Goryoukaku Hospital / Department of Pediatrics, Fukuoka University School of Medicine

| **Item** | **Topic** | **Content in the revised manuscript** |
| --- | --- | --- |
| **1** | **Title** | A Suspected Mosaic 3q29 Deletion in a Patient with Autism Spectrum Disorder and Multiple Micronutrient Deficiencies: A Case Report  The words “case report” appear in the title. The principal finding is stated as suspected rather than established, consistent with the fact that the copy number state was not verified and no independent confirmatory method was applied. |
| **2** | **Key Words** | 3q29 deletion; chromosomal mosaicism; chromosomal microarray analysis; RNF168; NBN; food selectivity; micronutrient deficiency; case report  Eight keywords. “Microdeletion syndrome” has been replaced by “3q29 deletion” and “chromosomal mosaicism”, so that the keywords do not assert a diagnosis that the data do not establish. |
| **3a** | **Abstract – Introduction** | 3q29 deletion syndrome (MIM #609425) is a rare genomic disorder with variable expressivity and incomplete penetrance. Somatic mosaicism for a 3q29 deletion in an affected proband has rarely been documented, and mosaicism inferred from attenuation of the array signal requires confirmation by an independent method. Where a deletion encompasses an autosomal recessive (AR) disease gene, evaluation of the second allele may be informative. Extreme food selectivity in autism spectrum disorder (ASD) carries substantial but underrecognized nutritional risk. |
| **3b** | **Abstract – Patient concerns and findings** | An 8-year-old Japanese boy with ASD, intellectual disability, absent speech, progressive obesity, hepatosplenomegaly and congenital toe syndactyly. Chromosomal microarray analysis (CMA) segmented a 1.27 Mb interval at 3q29 (arr[GRCh37] chr3:195,804,728–197,074,949) with a mean log2 ratio of −0.252 across 59 informative probes, against an excellent array quality (derivative log ratio spread 0.179). The attenuation is intermediate between a normal diploid state and that expected for a constitutional heterozygous deletion and is compatible with a suspected mosaic deletion; the laboratory’s automated nomenclature assigned a copy number of 2 with the explicit caveat that copy number was not verified, and no independent confirmatory method was applied.  Additional findings: microcytosis and hypochromia at both time points, with a serum iron within the reference interval at baseline and low at follow-up and a ferritin within the reference interval throughout, so that no threshold-based criterion for iron deficiency was met; low 25-hydroxyvitamin D; low vitamin C; low retinol-binding protein; a marginal elevation of PIVKA-II, measured only at follow-up. An incidental heterozygous NBN variant of uncertain significance was identified. |
| **3c** | **Abstract – Diagnoses, interventions, outcomes** | Working formulation: an attenuated CMA signal at 3q29 compatible with a suspected mosaic deletion, in a patient whose phenotype overlaps 3q29 deletion syndrome. The copy number state and the mosaic fraction remain unverified.  Investigation: targeted 15-gene DNA repair panel (PID_DDR_v1, Kazusa DNA Research Institute) identified no pathogenic or likely pathogenic RNF168 variant within the analytical scope of the panel, providing no molecular support for compound heterozygosity or RIDDLE syndrome. The incidental heterozygous NBN c.265A>G (p.Ile89Val) variant of uncertain significance was non-diagnostic and did not alter clinical management.  Interventions: oral vitamin A 10,000 IU/day; oral iron; dietary counselling to increase vitamin C intake; a vitamin D supplementation plan; referral to an obesity outpatient clinic and to a registered dietitian experienced in ASD-associated feeding difficulties.  Outcome: repeat laboratory testing approximately five months later showed persistent biochemical abnormalities, and the obesity index rose from 61% to 71.2%. No vitamin D had been administered before the follow-up sample, and the duration of and adherence to the other supplements were not documented, so these findings do not establish that supplementation failed. The parents reported some broadening of accepted foods; this was not corroborated by objective data. |
| **3d** | **Abstract – Conclusion** | Three take-away messages: (1) an attenuated CMA signal is compatible with, but does not establish, a mosaic deletion, and should be reported as suspected until confirmed by an independent method; (2) where a deletion encompasses an AR disease gene whose phenotype overlaps the presentation, or where that disorder is clinically actionable, second-allele evaluation may add diagnostic resolution; (3) this case highlights the importance of considering nutritional assessment in children with ASD and extreme food selectivity, the abnormalities having persisted at follow-up while the underlying dietary restriction was unchanged. |
| **4** | **Introduction** | Section 1. Establishes: the clinical spectrum, variable expressivity and incomplete penetrance of 3q29 deletion syndrome, including the possibility of inheritance from an unaffected or mildly affected parent; the basis on which mosaicism is inferred from array data and why an attenuated signal alone is insufficient; the rarity of reported mosaicism at this locus, the only documented instance being an unaffected mosaic father confirmed by quantitative PCR; and the presence of RNF168 (OMIM #612688) within the region, with RIDDLE syndrome (MIM #611943) reported in only four patients worldwide.  References cited: Willatt et al. 2005; Cox & Butler 2015; Petrin et al. 2011; Stewart et al. 2007, 2009; Devgan et al. 2011; Pietrucha et al. 2017. |
| **5a** | **Patient information – de-identified** | 8-year-old Japanese male, the fifth child of healthy, non-consanguineous parents. Born at 40 weeks and 1 day by spontaneous vaginal delivery (birth weight 3,204 g; length 51 cm; head circumference 33 cm). Unremarkable neonatal period. No name, date of birth, hospital identifier, dates of individual encounters or images of the face are included anywhere in the manuscript, tables or figure. |
| **5b** | **Patient information – primary concerns and symptoms** | Progressive weight gain from age 4 years (approximately 10 kg over 12 months); extreme food selectivity with a lifetime repertoire of fewer than ten foods; absent speech at any stage; developmental delay identified in the preschool years; hepatosplenomegaly identified on screening ultrasonography. |
| **5c** | **Patient information – medical, family, psychosocial and genetic history** | Medical: ASD diagnosed in the preschool years at another institution, with the diagnostic instruments unavailable for review; intellectual disability characterized as severe on the basis of parental report and clinical observation, formal psychometric testing not performed; congenital syndactyly of the second and third toes of the right foot, surgically corrected at 9 months.  Family: parents healthy and non-consanguineous; the patient is the fifth child; one sibling has short stature. Parental samples were not obtained and parental CMA could not be performed; the reason is not recorded in the clinical notes, family testing having been raised for discussion without a documented decision or declinature.  Psychosocial: attends a special-support school; unable to participate in standardized cognitive assessment.  Genetic: arr[GRCh37] 3q29(195804728_197074949) — attenuated signal, copy number not verified, compatible with a suspected mosaic deletion; arr[GRCh37] 9p13.1p12(38869901_42416387)x3; arr[GRCh37] 11p15.3p15.2(12051354_12916367)x3; incidental heterozygous NBN c.265A>G (p.Ile89Val) variant of uncertain significance. All coordinates are given against GRCh37/hg19, consistent with the main text and Table 2. |
| **5d** | **Patient information – past interventions** | Syndactyly repair at 9 months of age, with a satisfactory result. Behavioral approaches to food selectivity over several years, without expansion of the dietary repertoire. Ongoing ASD-related educational and behavioral support. |
| **6** | **Clinical findings** | Initial assessment (August 2025): height 124.4 cm (−0.34 SD), weight 38.8 kg (+1.97 SD), BMI 25.1 kg/m² (+2.25 SD), obesity index 61%. Most recent review (February 2026, approximately six months after the initial assessment): height 127.4 cm (−0.29 SD), weight 43.9 kg (+2.1 SD), BMI 27.0 kg/m² (+2.3 SD), obesity index 71.2%.  No thyroid enlargement, striae, acanthosis nigricans or single palmar crease. Increased lanugo-like hair density in the mandibular region.  Detailed dysmorphological assessment, including the craniofacial features characteristic of 3q29 deletion syndrome, was not undertaken because consent for documentation of facial features was not obtained; their presence or absence is therefore not commented upon.  Abdominal ultrasonography (November 2025): hepatomegaly (left lobe 153.1 mm, upper limit for age 130.1 mm; right lobe 113.4 mm, upper limit 105.5 mm) with mildly increased parenchymal echogenicity; splenomegaly (spleen index 37.3; upper limit for ages 6–8 years 28.9); gallbladder, common bile duct and kidneys normal; pancreas not adequately visualised; measurements approximate because respiratory gating was not possible. Urinalysis normal. |
| **7** | **Timeline** | Birth: 40 weeks + 1 day; birth weight 3,204 g; unremarkable neonatal period.  9 months: surgical correction of right-foot toe syndactyly.  Preschool years: ASD diagnosed elsewhere; absent speech; developmental delay.  Age 4 years: onset of progressive weight gain; longstanding food selectivity becomes a clinical concern.  Several years: behavioural approaches to food selectivity, without expansion of the repertoire.  Age 8 years, initial assessment (August 2025): referral for progressive obesity; anthropometry; baseline laboratory and nutritional screen; plasma amino acids; CMA requested.  Age 8 years, two weeks later (August 2025): CMA result reviewed; targeted 15-gene DNA repair panel planned; supplementation of vitamin A and iron commenced, with a vitamin D plan established but no dose administered.  Age 8 years (November 2025): abdominal ultrasonography.  Age 8 years (January 2026, approximately five months after the initial assessment): repeat laboratory panel; immunoglobulins and AFP; DNA repair panel result.  Age 8 years (February 2026, approximately six months after the initial assessment): most recent clinical review and anthropometry. The laboratory follow-up and the anthropometric review are separate time points and are not reported as a single interval.  Presented as a narrative timeline in Section 2, where the same months and intervals are used; no separate timeline figure is included. |
| **8a** | **Diagnostic assessment – methods** | Physical examination with anthropometry; abdominal ultrasonography; urinalysis.  CMA: Agilent GenetiSure Dx Postnatal platform, GenetiSure Dx Postnatal Analysis Method_v1, referenced to GRCh37/hg19 against an Agilent Male Reference; derivative log ratio spread 0.179. Probe-level data and array visualizations were obtained from the laboratory (SRL, Inc., Tokyo, Japan).  Targeted 15-gene DNA repair panel (PID_DDR_v1; Kazusa DNA Research Institute): ATM, MRE11, NBN, RAD50, LIG4, NHEJ1, DCLRE1C, PRKDC, DNMT3B, ZBTB24, CDCA7, HELLS, RNF168, MCM4, BLM. The panel covers coding exons and flanking intronic sequence and does not reliably detect deep intronic variants, regulatory variants or intragenic copy number change.  Laboratory: full blood count; iron studies with ferritin and CRP; retinol-binding protein; 25-hydroxyvitamin D; vitamin C; PIVKA-II; vitamins B1, B2 and B12; zinc, copper, selenium and magnesium; serum carnitine fractions; plasma amino acid analysis; immunoglobulins and alpha-fetoprotein; liver and renal function; lipids; somatomedin-C.  Not performed: fluorescence in situ hybridization, quantitative PCR, multiplex ligation-dependent probe amplification or droplet digital PCR; exome or genome sequencing (neither is reimbursed for this indication under the Japanese national health insurance system, whereas the targeted DNA repair panel including RNF168 was a covered investigation); parental CMA; transferrin saturation and total iron-binding capacity; hemoglobin electrophoresis and hemoglobin A2 quantification; serum retinol; coagulation studies; ceruloplasmin and urinary copper; portal Doppler assessment; lysosomal enzyme assays; functional or cytogenetic testing of cellular radiosensitivity. PIVKA-II was measured only at follow-up; plasma amino acid analysis only at the initial assessment. |
| **8b** | **Diagnostic assessment – challenges** | The central difficulty is that an attenuated array signal is compatible with a mosaic deletion but does not establish one. The plots, the probe-level values and the segment-boundary observation all derive from the same experiment. The laboratory’s automated nomenclature assigned a copy number of 2 with the explicit caveat that copy number was not verified, and confirmatory testing by an independent method was not feasible in this clinical setting.  Adjacent array probes within a segmented interval are not independent biological observations; probe-level values are therefore reported descriptively and no inferential statistical testing of the segment mean was undertaken.  Severe intellectual disability precluded standardized cognitive testing; consent for documentation of facial features was not given, so the craniofacial phenotype could not be assessed; and the etiology of the obesity (dietary versus genetic) could not be resolved. |
| **8c** | **Diagnostic assessment – diagnoses** | Principal finding: an attenuated CMA signal at arr[GRCh37] 3q29(195804728_197074949), 1,270.222 kb, 59 informative probes, mean log2 −0.252, automated copy number call x2 with copy number not verified. Reported as compatible with a suspected mosaic 3q29 deletion; not independently confirmed. The interval is atypical and nested within the recurrent 3q29 region and contains TFRC, RNF168, PAK2 and DLG1.  RIDDLE syndrome (MIM #611943): no pathogenic or likely pathogenic RNF168 variant was detected within the analytical scope of the panel, providing no molecular support for compound heterozygosity. The manuscript does not state that RIDDLE syndrome has been excluded. Immunoglobulins and AFP were normal; cellular radiosensitivity was not tested and is described as unassessed rather than absent.  Incidental: heterozygous NBN c.265A>G (p.Ile89Val; NM_002485.5; rs61754966), variant of uncertain significance (ClinVar: conflicting interpretations); Japanese ToMMo allele frequency 0.00584, supporting ACMG criterion BS1. Non-diagnostic; did not alter management.  Nutritional findings, stated descriptively: microcytosis and hypochromia with a low serum iron at follow-up, a pattern compatible with but not confirming iron deficiency (the hemoglobin concentration does not meet criteria for anemia at this age; serum iron and ferritin were within the reference intervals at baseline, ferritin did not fall below the World Health Organization threshold of 15 µg/L, and transferrin saturation, total iron-binding capacity, hemoglobin electrophoresis and hemoglobin A2 quantification were not measured; the Mentzer index was above 13 at both time points, favoring iron deficiency over beta-thalassemia trait, but the red-cell count was at or above the upper reference limit at both time points, so a thalassemia trait cannot be excluded); 25-hydroxyvitamin D in the deficient range; low vitamin C; low retinol-binding protein, a surrogate marker on which a diagnosis of vitamin A deficiency cannot be established because serum retinol was not measured; a marginal elevation of PIVKA-II, measured only at follow-up, suggestive of possible subclinical vitamin K insufficiency.  Other: ASD; intellectual disability; hepatomegaly with increased parenchymal echogenicity and disproportionate splenomegaly, cause unresolved.  Other copy number findings: arr[GRCh37] 9p13.1p12(38869901_42416387)x3, benign; arr[GRCh37] 11p15.3p15.2(12051354_12916367)x3, variant of uncertain significance, likely benign. Both are described as gains, not as hemizygous. All coordinates are given against GRCh37/hg19. |
| **8d** | **Diagnostic assessment – prognostic characteristics** | No mosaic fraction is asserted. Applying log2((2 − f)/2) to the observed mean yields f ≈ 32%; allowing for the signal compression typical of array CGH gives 36–50%. These figures, spanning approximately 32–50%, are platform- and algorithm-dependent inferences from signal intensity, not a direct measurement of the proportion of abnormal cells, and the mosaic fraction was not independently determined. No inference is drawn about other tissues.  No prognostic statement is made on the basis of the NBN variant of uncertain significance, and no oncological surveillance is recommended. Any decision regarding surveillance would rest on the patient’s personal and family history.  Hepatomegaly with increased echogenicity and disproportionate splenomegaly remain unexplained and are not attributed to the 3q29 finding; further investigation is recommended.  Parental samples were not obtained — the reason is not recorded in the clinical notes — so the de novo versus inherited origin is undetermined and recurrence-risk counselling remains incomplete. |
| **9a** | **Therapeutic intervention – types** | Pharmacological: oral vitamin A; oral iron; a vitamin D supplementation plan.  Nutritional and behavioral: dietary counselling to increase vitamin C intake; referral to a registered dietitian experienced in ASD-associated feeding difficulties.  Referrals and preventive care: ophthalmological, dental and cardiac evaluation; obesity outpatient clinic.  The family independently commenced a commercial sulforaphane supplement; this was not prescribed. |
| **9b** | **Therapeutic intervention – administration** | Vitamin A: 10,000 IU/day orally.  Iron: oral supplementation; the dose is not recorded in the clinical record and is therefore not stated in the manuscript.  Vitamin D: a supplementation plan was established; no dose had been administered by the time of the follow-up sample.  Vitamin C: dietary counselling only, delivered through commercially available vitamin C-fortified confectionery, this being the only vehicle the patient would reliably accept; no supplement dose was prescribed.  Dietitian input: referral made; the number of sessions and the specific behavioural approach were not systematically documented. |
| **9c** | **Therapeutic intervention – changes** | No changes to the interventions were made during the reported period. Ophthalmological, dental and cardiac evaluations were pending at the time of reporting. |
| **10a** | **Follow-up and outcomes – outcomes** | Biochemical (January 2026, approximately five months after the initial assessment): repeat testing showed persistent abnormalities. Retinol-binding protein 2.0 → 1.8 mg/dL; 25-hydroxyvitamin D 15.2 → 9.7 ng/mL (no vitamin D had been administered before the second sample, and the samples were drawn in late summer and midwinter respectively); vitamin C 1.9 → 2.8 µg/mL, remaining below the reference range; serum iron 75 → 30 µg/dL, with a rise in ferritin confounded by a concurrent rise in CRP; AST 27 → 47 U/L and ALT 16 → 35 U/L.  Treatment exposure: the duration of, and adherence to, vitamin A and iron supplementation were not systematically documented, and no vitamin D was given before the follow-up sample. The persistence of these abnormalities is therefore reported as an observation and is not presented as failure of supplementation.  Anthropometric (February 2026, approximately six months after the initial assessment, and therefore a later time point than the laboratory follow-up): obesity index 61% → 71.2%; BMI 25.1 → 27.0 kg/m² — that is, continued weight gain.  Dietary: the parents reported some broadening of accepted foods following the dietitian referral. The number and types of newly accepted foods, the duration of follow-up and any structured measure of dietary intake were not documented. This is a parental report only, is not corroborated by the biochemical or anthropometric data, and is described in the manuscript as anecdotal. No claim of treatment success and no claim of priority is made. |
| **10b** | **Follow-up and outcomes – follow-up tests** | Performed: repeat full blood count, iron studies with ferritin and CRP, liver function, retinol-binding protein, 25-hydroxyvitamin D, vitamin C, PIVKA-II, vitamins B1, B2 and B12, zinc, copper, selenium, magnesium, carnitine fractions, immunoglobulins, alpha-fetoprotein and somatomedin-C; repeat anthropometry.  Recommended but not yet performed: ceruloplasmin with 24-hour urinary copper; portal Doppler ultrasonography; lysosomal enzyme assays; repeat abdominal ultrasonography; orthogonal confirmation of the 3q29 finding; parental CMA. |
| **10c** | **Follow-up and outcomes – adherence and tolerability** | Adherence to oral vitamin A was reported by the parents; no structured adherence measure was applied. The patient is able to swallow tablets. No formal assessment of adherence to the dietary counselling was undertaken, and this is stated as a limitation. |
| **10d** | **Follow-up and outcomes – adverse events** | No adverse or unanticipated events were reported. |
| **11a** | **Discussion – strengths and limitations** | Strengths: complete transparency of the array data, including probe-level values, array visualizations and the laboratory’s own nomenclature and caveat; a defined rationale for second-allele evaluation of an AR disease gene contained within the interval; and a broad, paired nutritional and metabolic characterization.  Limitations, as stated in the manuscript: the 3q29 finding rests on a single CMA experiment and was not independently confirmed; the copy number state remains unverified; the mosaic fraction is an inference from signal intensity and was not independently determined; parental samples were not obtained; exome or genome sequencing was not performed, neither being reimbursed for this indication; the estimate applies to peripheral blood leucocytes only; dysmorphological assessment was not undertaken; the ASD diagnosis was made elsewhere and intellectual disability was not formally graded; follow-up comprised two time points over approximately five months and the reported dietary improvement rests on parental report alone; treatment exposure and adherence were not documented and no vitamin D was administered before the follow-up sample; PIVKA-II was measured only at follow-up; cellular radiosensitivity was not assessed; and transferrin saturation, total iron-binding capacity, hemoglobin electrophoresis, hemoglobin A2 quantification, serum retinol, coagulation studies, ceruloplasmin, urinary copper, portal Doppler assessment and lysosomal enzyme assays were not undertaken. |
| **11b** | **Discussion – medical literature** | 3q29 phenotype, variable expressivity and incomplete penetrance: Willatt et al. 2005; Cox & Butler 2015.  Mosaicism at this locus: Petrin et al. 2011 (an unaffected mosaic father, confirmed by quantitative PCR).  RNF168 and RIDDLE syndrome: Stewart et al. 2007, 2009; Devgan et al. 2011; Pietrucha et al. 2017.  Food selectivity in ASD: Geraghty et al. 2010. Vitamin D and ASD: Cannell 2017.  Pericentromeric 9p euchromatic variants: Lecce et al. 2006; Willatt et al. 2007.  The reference formerly cited in support of oncological surveillance has been removed together with that recommendation. |
| **11c** | **Discussion – rationale** | An attenuated array signal, however internally consistent, cannot establish a mosaic deletion; the finding is therefore reported as suspected throughout, and the reasoning behind that position is set out explicitly rather than asserted.  Second-allele evaluation is proposed as a considered step where a deletion encompasses an AR disease gene whose phenotype overlaps the presentation, or where that disorder is clinically actionable — not as a routine step in every such case. Where the phenotype remains incompletely explained, broader sequencing is the more comprehensive investigation.  Concurrent abnormalities across several micronutrient pathways may accumulate silently in extreme food selectivity. A single case cannot establish the value of systematic screening, and no such claim is made; the manuscript states only that this case highlights the importance of considering nutritional assessment in this population. |
| **11d** | **Discussion – take-away lessons (Conclusion)** | “In conclusion, an attenuated CMA signal at 3q29 was identified in an 8-year-old boy with ASD and intellectual disability. The magnitude of the attenuation is compatible with a suspected mosaic deletion, but the finding was not confirmed by an independent method and the copy number state remains unverified; it should not be regarded as establishing a mosaic deletion. Where a deletion encompasses an AR disease gene whose phenotype overlaps the presentation, evaluation of the second allele may add diagnostic resolution; where the phenotype remains incompletely explained, broader sequencing should be considered. Extreme ASD-associated food selectivity was accompanied by concurrent abnormalities across several micronutrient pathways, which highlights the importance of considering nutritional assessment in children with ASD and extreme food selectivity. The hepatosplenomegaly remained unexplained after the investigations reported here and is not attributed to the 3q29 finding.”  A single paragraph, without references, and containing no absolute expressions such as “definitively excluded”, “definitive genetic clarity” or “first documented”. |
| **12** | **Patient perspective** | The patient has severe intellectual disability and no speech and cannot give a perspective in his own words. No formal written statement was obtained from the parents. The manuscript therefore contains no quoted patient or parental perspective, and the paragraph previously presented as a parental perspective has been removed.  The only parent-derived observation retained is the report of some broadening of accepted foods, which appears in Section 2.6 and in the limitations, is explicitly attributed to parental report, and is described as anecdotal and uncorroborated. |
| **13** | **Informed consent** | “This case report was approved by the Institutional Review Board of Hakodate Goryoukaku Hospital (approval number: 2025-062; date of approval: March 17, 2026). Written informed consent for publication was obtained from the patient’s parents.” (Section 2.1)  Consent for documentation of facial features was specifically not given, and no facial image or dysmorphological description appears anywhere in the manuscript.  The signed consent form is uploaded separately as confidential supplementary material, not for review and not for publication, in accordance with the journal’s instructions. |
